# Synergistic Hydrothermal Conversion of Aqueous Solutions of CO_2_ and Biomass Waste Liquefaction into Formate

**DOI:** 10.1021/acssuschemeng.2c06218

**Published:** 2022-12-02

**Authors:** María Andérez-Fernández, Eduardo Pérez, Ángel Martín, James McGregor, María Dolores Bermejo

**Affiliations:** † Grupo de Tecnologías a Presión (PressTech), Instituto de Bioeconomía de la Universidad de Valladolid (BioEcoUVa), Departamento de Ingeniería Química y Tecnologías del Medio Ambiente, Escuela de Ingenierías Industriales, Universidad de Valladolid, 47011Valladolid, Spain; ‡ Departamento de Química Física, Facultad de Químicas, Universidad Complutense de Madrid. Avda Complutense s/n, 28040Madrid, Spain; § Department of Chemical and Biological Engineering, University of Sheffield, SheffieldS1 3JD, U.K.

**Keywords:** NaHCO_3_, sugarcane bagasse, pine needles, CO_2_ reduction, biomass valorization, subcritical water, acetic acid, lactic acid

## Abstract

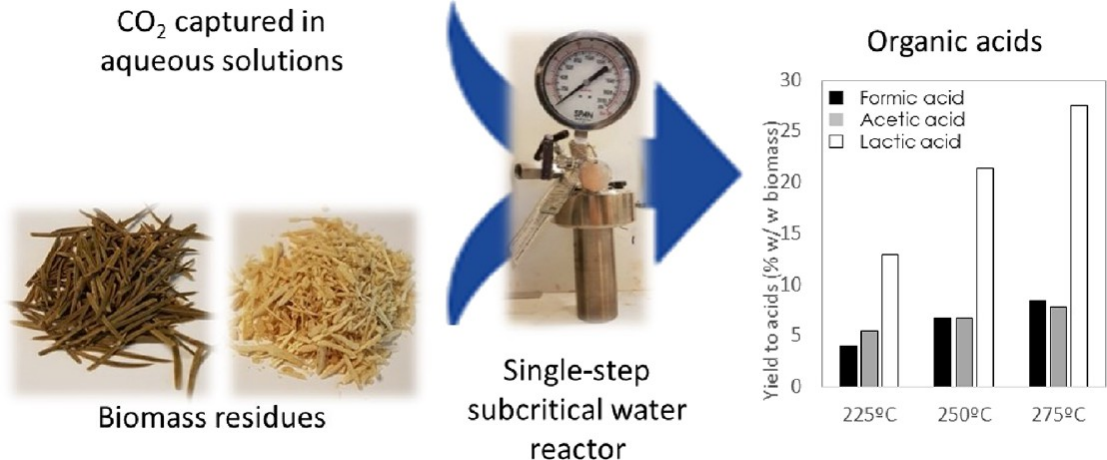

CO_2_ utilization by conversion into useful chemicals can contribute to facing the problem of increasing CO_2_ emissions. Among other alternatives, hydrothermal transformation stands out by the high conversions achieved, just using high-temperature water as the solvent. Previous works have demonstrated that several organic compounds with hydroxyl groups derived from biomass can be used as reductants of NaHCO_3_ aqueous solutions as inorganic CO_2_ sources. Formate was obtained as the main product as it was produced by conversion both of the inorganic carbon and of the organic reductants, whose transformation into formate was promoted by the addition of NaHCO_3_. Based on these results, in this work, the hydrothermal conversion of NaHCO_3_ is performed together with the liquefaction of lignocellulosic biomass (sugarcane bagasse and pine needles) in a one-pot process. Results show that yields to formate of 10% wt/wt (with respect to the initial concentration of biomass) are achieved by hydrothermal treatment of NaHCO_3_ and lignocellulosic biomass at 250 °C with a residence time of 180 min. Other products, such as acetic acid and lactic acid, were also obtained. These results demonstrate the feasibility of the hydrothermal reduction of CO_2_ combined with the hydrothermal liquefaction of residual biomass in a simultaneous process.

## Abstract 2

Formic, acetic, and lactic acids are produced by means of simultaneous hydrothermal CO_2_ and biomass wastes (sugarcane bagasse and pine needles) valorization.

## Introduction

CO_2_ capture, conversion, and utilization have been researched in the past years as a method to reduce CO_2_ concentration in the atmosphere and obtain valuable and useful products.^1^ Among the different technologies for CO_2_ conversion, hydrothermal reduction (using water at high temperatures, ∼200–350 °C, and pressures above saturation, 15–160 bar) presents some promising advantages; the most remarkable of these is the use of only water as the solvent and hydrogen donor.^2^ In this way, H_2_ gas utilization is avoided, resulting in a safer process and a lower dependence in fossil fuels for its generation.^3^ Moreover, this process uses alkaline aqueous solutions of CO_2_ (e.g., dissolved as NaHCO_3_) as feedstock, which facilitates the integration with the capture of CO_2_ by absorption with alkaline aqueous solutions (e.g., of NaOH or alkanolamines), which currently is the most technically developed method for CO_2_ capture at the industrial scale. With this integration, the aqueous solutions of CO_2_ that are produced by current industrial CO_2_ capture processes and that without further processing constitute waste whose disposal, e.g., by geological sequestration, is currently problematic, can be valorized into useful products.

Previous studies have shown the capacity of different organic molecules to act as a CO_2_ reductant under hydrothermal conditions.^4,5^ A wide range of these molecules, in most cases obtained from lignocellulosic biomass, showed significant yields to formic acid (FA) when NaHCO_3_ was added to the reaction medium, reaching yields up to 90% in the case of C3 alcohols, such as isopropanol and glycerol.^6,7^ Further studies, including previous works of the authors, have investigated the origin of FA when using complex organic molecules, e.g., glucose and algae, as reductants, using marked NaH^13^CO_3_ as the CO_2_ source. These investigations showed that FA production resulted both from the NaH^13^CO_3_ reduction (as determined by ^13^C-NMR analyses) and from glucose decomposition.^8,9^ It was also observed that both processes were synergistic in the sense that while on the one hand glucose and other organic derivatives acted as NaHCO_3_ reductants, on the other hand the addition of NaHCO_3_, an oxidant, to the aqueous media enhanced the yield and especially the selectivity of the conversion of glucose to FA.^9^

Thus, the optimization of the reaction may result in a process that achieves high yields to FA from both sources. The interest in the production of FA lies in the important role that it can play in the green hydrogen energy economy. FA is a potential liquid organic hydrogen carrier, which can be dehydrogenated or directly used as feedstock for power cells, resulting in a promising alternative to energy generation from fossil fuels. In addition, FA is an important commodity for different industries, such as textile, pharmaceutical, rubber, and agricultural industries, among others.^10^

These previous works with model organic substances have demonstrated the technical feasibility of the process, but in order to design a competitive and economic process of FA production, the reagents and feedstock costs should represent a minor fraction of the total disbursement of the process. Indeed, in a previous work of the authors in which an economic analysis of the hydrothermal conversion process is presented, considering a metal instead of biomass derivatives as a reductant, it was concluded that the reductant amounted for more than 50% of the production costs;^11^ therefore, the substitution of the expensive metal reductant by an inexpensive and renewable reductant such as organics derived from the liquefaction of biomass would greatly contribute to the economic feasibility of the process. Thus, the next required step is using lignocellulosic biomass directly as a reductant as it is a globally available, sustainable, and inexpensive feedstock, which can be obtained from other industries as their residues. Preferentially, second-generation feedstocks such as non-food crops or residual biomass should be used to avoid interference with food security or a negative environmental impact.^12^ Lignocellulosic biomass is mainly composed of cellulose, hemicellulose, and lignin, fractions that are closely linked, making its effective separation the main challenge of biorefinery with the aim of the valorization of biomass and conversion to structurally simpler continuouss.^13,14^

Different techniques have been proposed for the fractionation and valorization of biomass, being hydrothermal methods a highly promising alternative, due to the unique and advantageous properties of hot compressed water.^15,16^ For the direct transformation of biomass into formic acid, Shen et al.^17^ have established that the catalytic oxidative transformation is the most favorable process from the point of view of sustainability. Sahoo et al.^18^ have reviewed recent developments in this approach, and Jin et al.^19^ have demonstrated the conversion of glucose as a model compound of biomass into formic acid with conversion yields as high as 75%. However, this approach cannot be combined with the hydrothermal conversion of aqueous solutions of CO_2_ as the oxidant would prevent the reduction of NaHCO_3_. In contrast, direct hydrothermal liquefaction of lignocellulosic biomass without addition of any oxidative agent allows its conversion into a mixture of useful reducing chemicals (sugars, C2–C3 alcohols and aldehydes, phenolic compounds, etc.) in a one-pot reaction.^20,21^ Although the detailed mechanism of the reaction is complex and not fully understood, it has been established that this conversion proceeds through the hydrolysis of cellulose and hemicellulose into free sugars at short reaction times of even less than 1 s and continues through the further decomposition of these sugars into compounds such as glyceraldehyde, glycoladehyde, or 5-(hydroxymethyl) furfural at longer reaction times.^13,14^ Thus, it is possible to produce organic reductants for NaHCO_3_, e.g.*,* sugars and alcohols, in the same media in which the hydrothermal CO_2_ reduction is carried out. Moreover, several studies have reported that the addition of carbonates (CO_3_^2–^, which is in equilibrium with HCO_3_^–^ thereby increasing the pH of the solution) enhances the yield to liquid products in the liquefaction of biomass.^21−24^ In the presence of NaHCO_3_, these reducing organic compounds are oxidized to organic acids, while NaHCO_3_ is converted as well into formate, thus promoting the global selectivity of the process toward acids.^9^

Considering these antecedents, in this work, the hydrothermal liquefaction of lignocellulosic biomass waste samples (instead of the pure model organic compounds considered in previous studies) to organic compounds (particularly FA) and the hydrothermal reduction of NaHCO_3_ (as a CO_2_ source) to FA are performed simultaneously in a single process without intermediate steps of fractionation and purification. With this combination, the cumbersome and expensive intermediate steps of purification and separation of the organic reductants are avoided, and the use of energy is optimized because intermediate cooling and heating steps are also suppressed. Therefore, this work aims to experimentally demonstrate the feasibility of obtaining FA and other value-added chemicals from NaHCO_3_ and biomass treated simultaneously by hydrothermal processing. For this purpose, two types of biomass, pine needles and sugarcane bagasse, with different compositions and structural properties, are tested, and experiments are carried out at different temperatures and time conditions, with or without addition of NaHCO_3_.

## Experimental Section

### Materials

NaHCO_3_ (99%) was purchased from Across Organics. As lignocellulosic biomass samples, two different types of residues were used: pine needles (*Picea abies*) and sugarcane bagasse (using two particle sizes, 200–500 μm and powder). Both residues were dried over night at 105 °C. Additional information about other reagents used for analyses is provided as Supporting Information.

### Hydrothermal NaHCO_3_ Conversion and Biomass Liquefaction

Reactions were performed in a 100 mL batch reactor (Parr Instruments) using a magnetic stirrer bar (IKA C-MAG HS 7). Biomass was soaked overnight in 25 mL of water. Afterward, it was placed into the reactor with another 25 mL of NaHCO_3_ aqueous solution. The reactor was sealed and purged with a continuous flow of nitrogen for 5 min. The reactor was then heated by placing it in a preheated heating block at 350 °C, setting this instant as the initial time (*t* = 0 min). The desired temperature was reached after ∼10 min. At the end of the experiment, the reactor was cooled to room temperature and opened. The solid fraction was separated from the liquid phase by filtration and then washed with water and dried overnight in an oven at 70 °C. In order to ensure the reproducibility of the results, experiments were repeated twice, with a relative standard deviation lower than 10% in all cases.

### Liquid-Phase Analyses

Liquid samples were analyzed by HPLC using an Aminex 87H column (BioRad) set up in an HPLC separation module (Waters, Alliance module e2695). Two detectors were used, RI detector (Waters, 2414 module) and UV module, using a wavelength of 210 nm (Water, 2998 module). The total organic carbon (TOC) was determined with a TOC-VCSH instrument (Shimadzu).

Yields to organic products (formic acid, acetic acid, and lactic acid), *Y_i_*, referred to the initial amount of biomass, were calculated as expressed in eq 1:
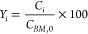
1where *C_i_* is the product concentration at the end of the reaction and *C*_*BM,*0_ is the initial concentration of biomass.

The relative standard deviation (RSD) for every experimental point was calculated as shown in eq 2:
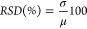
2where *RSD* is the relative standard deviation expressed in %, σ is the standard deviation, and μ is the calculated average of the set of values.

The yield to organic matter was defined as the proportion of organic carbon in solution (determined by TOC analysis) to the carbon content in the starting biomass as determined by elemental microanalysis (eq 3):
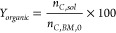
3where *n_C,sol_* and *n*_*C,BM,*0_ are the moles of organic carbon atoms in the liquid sample and starting biomass (determined by TOC analysis), respectively.

Selectivity to the identified acids (*S_i_*) was defined as the proportion of their concentration in the dissolved organic matter, also expressed in carbon mole percent, as shown in eq 4:
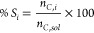
4where *n_C,i_* is the moles of carbon atoms in compound *i* and *n_C,sol_* is the total moles of organic carbon dissolved in the aqueous product.

The total acid selectivity is defined as the sum of selectivity for the three main acids obtained: formic, acetic, and lactic (that under the alkaline conditions of the reaction media are present as the corresponding sodium salts), as indicated in eq 5:
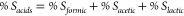
5

Other organic compounds in the solutions were identified to be mainly sugars and products from sugar degradation.

### Solid-Phase Analyses

Solid samples were recovered by filtration after reaction and dried overnight at 70 °C. Samples were afterward analyzed by scanning electron microscopy (SEM), Fourier-transform infrared spectroscopy (FT-IR), and CHNS elemental analysis. SEM analyses were carried out by coating the dried samples with gold for 10 s, applying 40 mA at a pressure of 0.04 mbar (Agar Sputter Coater) and then using a Jeol JSM-6010 LA analytical scanning electron microscope. The accelerating voltage employed was 5 kV. The functional groups of the solid sample were characterized by FT-IR (BRUKER, model ALPHA). FT-IR spectra were obtained at 4000–400 cm^–1^, with 4 cm^–1^ resolution and 64 scans. The elemental compositions of solid samples collected from biomass experiments were analyzed using an EA Flash 200 analyzer (Thermo Fisher Scientific) using a TCD detector and a microscale (Mettler Toledo XP6). The oven temperature was set at 900 °C, and the flows of gases were 140 mL/min of carrier (helium), 250 mL/min of oxygen, and 100 mL/min of helium.

## Results and Discussion

### Simultaneous and Synergistic Hydrothermal Biomass Liquefaction and NaHCO_3_ Reduction

**Figure 1:**
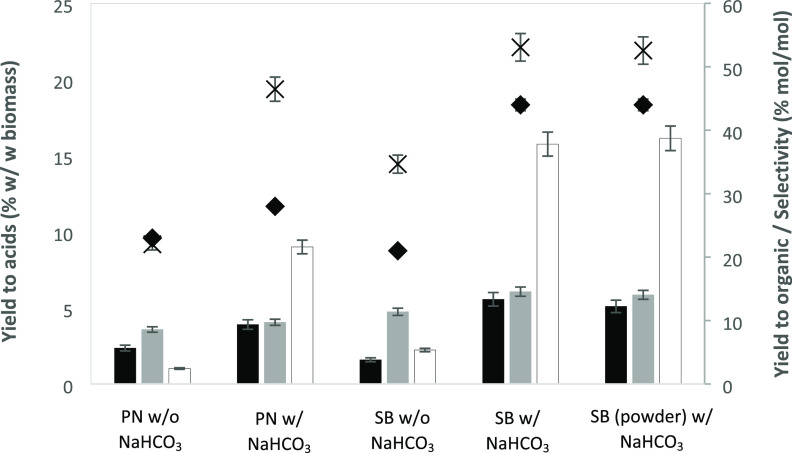
Effect of NaHCO_3_ addition on hydrothermal treatment of PN and SB. Reaction conditions: 20.0 g/L biomass, 250 °C, 30 min. Bars (left axis) represent yields: (gray bars): FA, (black bars): AA, and (clear bars): LA. Symbols (right axis): (cross): total yield of liquefied matter, %*Y*_organic_, and (diamonds): selectivity of the identified acids, %*S*_acids_, both expressed in mol of carbon. The rest of the dissolved organics are mostly sugars.

Both types of biomass, pine needles (PN), and sugarcane bagasse (SB) were treated hydrothermally with and without NaHCO_3_ at 250 °C for 30 min starting with an initial concentration of 20.0 g/L of biomass. The obtained yields are presented in Figure 1 along with the overall acid selectivity and the yield of liquefied matter. As presented in Figure 1, experiments with pine needles and NaHCO_3_ produced yields of about 5% formic acid, 5% acetic acid, and 10% lactic acid, while experiments with sugarcane bagasse produced 7% formic acid, 7% acetic acid, and 16% lactic acid. In comparison, yields obtained in previous works using pure model organic compounds instead of biomass as reductants^5^ produced yields that were up to 65% in the case of experiments with glucose under similar operating conditions (300 °C, 180 min of reaction time). The lower yields produced with the real biomass samples can be attributed to the hampering effect of using a solid biomass that must be liquefied before reacting with NaHCO_3_ instead of water-soluble glucose and to the complexity of the structure and chemical composition of the biomass: in the biomass samples, only the cellulose/hemicellulose fraction can be readily converted into sugars such as glucose, which can be further converted into FA, and the cellulose fraction represents only approximately 40% in the case of pine needles and 50% in sugarcane bagasse, values that set an upper limit in the conversion yields that can be achieved.

Hydrothermal liquefaction of PN and SB yielded variable quantities of mixtures of water-soluble organics. The main products were formic acid (FA), acetic acid (AA), and lactic acid (LA), which in experiments with NaHCO_3_ that set alkaline conditions are present in the solution as the corresponding salts (sodium formate, acetate, and lactate). As indicated in the product chromatogram provided as supplementary information (Figure S2), additional products include glyceraldehyde, glycolaldehyde, and furfural, which in previous works have been identified as reaction intermediates in the transformation of bicarbonate with sugars produced by hydrolysis of cellulose/hemicellulose to FA,^9^ small amounts of non-hydrolyzed glucose and fructose, and also small concentrations of ethanol.

The addition of NaHCO_3_ to the reaction led to a significantly higher production of formic, acetic, and, particularly, lactic acid. It can be expected that a fraction of the FA produced was formed from bicarbonate reduction, as observed in previous works with model compounds and algae, in which experiments using NaH^13^CO_3_ demonstrated by NMR analysis that up to 80% of FA was produced by CO_2_ conversion and the remaining proportion originated from biomass decomposition.^5,10,11^ In a previous work of the authors using glucose as a model compound of the hydrothermal decomposition of the cellulose fraction of biomass and tracking the conversion of inorganic NaH^13^CO_3_ by NMR,^12^ it was found that under the experimental conditions considered in this work (250 °C, NaHCO_3_ inorganic carbon source), 30% of the produced formic acid originated from the inorganic carbon source and the remaining amount was produced from glucose. The enhancement in the production of AA and LA by addition of NaHCO_3_ can be assigned to a change in the reactive environment and particularly the higher pH. This results in a faster decomposition of the biomass into sugars and therefore, extracted sugars might more easily be available to react with the bicarbonate ion. In turn, these result in a higher production of AA and LA due to the rupture of hemicelluloses into oligomers and monomers, with their subsequent reaction and degradation into the observed products.

Regarding the type of biomass tested, sugarcane bagasse gave better results than pine needles either in plain water or with NaHCO_3_ added, reaching a yield of matter in solution up to 23% and a selectivity of 45% for the combination of the three identified acids. These differences between biomass sources might be related to the differing composition in hemicelluloses, cellulose, and lignin of the feedstocks, but structural differences may also play a role. In further experiments, sugarcane bagasse was selected to optimize the reaction conditions.^25,26^ From a practical perspective, the addition of bicarbonate brings an additional advantage, namely the enhancement of the selectivity to the identified acids, which will simplify subsequent downstream purification stages. AA is an important commodity chemical, and LA is the main building block for renewable biodegradable polylactic acid.

In the case of the particle size of sugarcane bagasse, in the interval tested in experiments, this parameter did not affect the results as the reaction with powder (particle size: <200 μm) yielded similar results to reactions with 200–500 μm particles. Thus, the operational costs can be reduced by avoiding an exhaustive milling step, making the process more competitive.

### Influence of the Initial Amount of Sugarcane Bagasse and Reaction Temperature on the Yield to FA and Other Products

**Figure 2:**
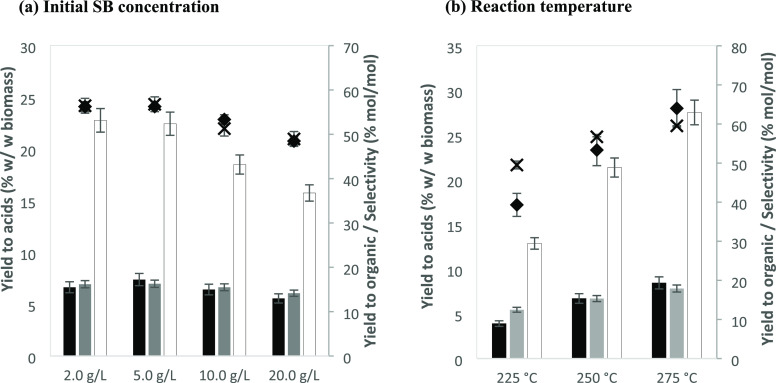
Yield to products as a function of (a) initial SB concentration (reaction conditions: 250 °C, 30 min, 42 g/L NaHCO_3_) and (b): reaction temperature (reaction conditions: 5.0 g/L sugarcane bagasse, 30 min, 42 g/L NaHCO_3_)**.** Bars (left axis): yield, expressed in (wt/wt) to (gray bars):FA, (black bars): AA, and (clear bars): LA. Symbols (right axis): (cross): total yield of liquefied matter, %*Y*_organic_, and (diamonds): selectivity of the identified acids, %*S*_acids_, both expressed in mol of carbon.

Experiments with different concentrations of SB (from 2.0 to 20.0 g/L) in 50 mL of water were carried out at 250 °C for 30 min, with an initial concentration of 42 g/L NaHCO_3,_ as shown in Figure 2A. In previous works, when using glucose as a reductant, it was observed that the higher the concentration, the lower the yield to FA.^7^ In this case, it is seen that the variation in the yield to FA and AA is not significant when increasing the initial amount of SB. The yield of FA varies merely from 6.6% at 2.0 g/L and 7.4% at 5.0 g/L to 5.6% at 20.0 g/L. In the case of LA, however, this effect is much more marked, decreasing from 22.8% at 2.0 g/L to 15.8% at 20.0 g/L. Similar results were obtained in the case of the yield of liquefaction and selectivity to products. It can be observed that a higher concentration of biomass resulted in a lower yield of liquefied matter and a lower selectivity to the main products, reaching a maximum of 56.8 and 56.3%, respectively, using an initial concentration of 5.0 g/L. Similarly to previous works using glucose as a reductant, a higher concentration of biomass in the reaction media may hinder the formation of FA due to the release of other carboxylic acids to the media, including AA, produced by the cleavage of acetyl groups linked to oligosaccharides that constitute the structure of biomass.^7,27−29^ Thus, the release of acetyl groups to the media may acidify it and shift the equilibrium of carbonate toward the acid species, H_2_CO_3_, thus inhibiting the formation of FA.

In the case of the reaction temperature, three different temperatures (225, 250, and 275 °C) were tested. An increase in the reaction temperature led to an improvement in the yield of the three acids (Figure 2B), particularly of LA. The yield to different products was doubled upon increasing the reaction temperature by 50 °C, raising from 3.9% at 225 °C to 8.5% at 275 °C in the case of FA and from 12.9% at 225 °C to 27.5% at 275 °C for LA. Moreover, an increase in the concentration of dissolved matter is observed as well as the increase in total acid selectivity. In the case of the yield to liquefaction, a positive effect of temperature can be observed, as yield increased from 49.5% at 225 °C to 59.5% at 275 °C. This effect is more pronounced in the case of the selectivity toward acids as it increased from 39.1% at 225 °C to 64.0% at 275 °C. The influence of the temperature on the production of liquefaction of biomass into the media is clearly observed as well as in the yield to the main carboxylic products obtained in the reaction. Previous works also showed that the temperature favored the reduction of NaHCO_3_ with glucose, obtaining FA as the product and AA and LA as by-products. In this case, higher reaction temperatures ease the solution of biomass into the liquid media, facilitating the reaction of sugars in the presence of NaHCO_3_ for the production of FA, AA, and LA.^7^

### Influence of Reaction Time

**Figure 3:**
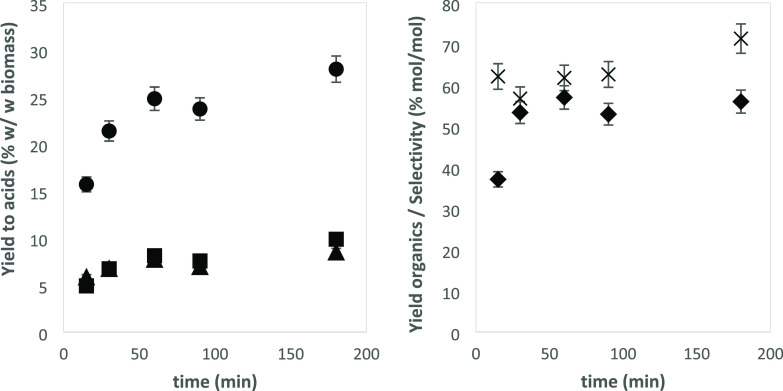
Effect of reaction time (from 15 to 180 min). Reaction conditions: 42.0 g/L NaHCO_3_, 5.0 g/L SB, 250 °C. Left graph: symbols: yield to (squares):FA, (triangles): AA, and (circles): LA. Right graph: (cross): total yield of liquefied matter, %*Y*_organic_, and (diamonds): selectivity of the identified acids, %*S*_acids_, both expressed in mol of carbon.

The reaction temperature 250 °C was selected to study the influence of the reaction time in the process. As presented in Figure 3, the production of FA increases with reaction time, reaching a maximum yield of 10% after 180 min of reaction. The same trend is observed for AA and LA, with final yields of 8.5 and 29.9%, respectively. However, the production rate of the three compounds was the fastest during the first 30 min of reaction, slowing down after this point. These results are similar to those obtained in previous works when using glucose as the model organic reactant, in which long reaction times prompted the production of FA, as well as the other main by-products.^12^ Differently to the results obtained in previous works with glucose as the reactant in which at short reaction times, yields to FA and other acids were high (yields that through experiments with marked NaH^13^CO_3_ were ascribed to the conversion of glucose^12^), in this case, at short times (*t* = 15 min), yields and selectivity toward the main products (FA, AA, and LA) were low at short reaction times, increasing afterward the yield and selectivity to products.^7^ These changes may be related to the complexity of the biomass as it is strongly entangled. These results indicate that when using unfractionated biomass, the reaction proceeds in two stages: first, liquefaction of biomass and second, formation of acids from the dissolved compounds, with the latter step exhibiting a lower rate. During the first stage, as biomass has a recalcitrant structure, time and temperature are required in order to dissolve the different fractions into the water (especially hemicellulose), producing low quantities of carboxylic acids. Longer reaction times prompt the dissolution of the fractions and the cleavage of the monomers, producing then carboxylic acids as products of reaction with NaHCO_3_.

### Influence of the Alkaline Medium in the Reaction

To further discriminate between the direct influence of NaHCO_3_ on the formation of FA and the indirect influence of NaHCO_3_ on the conversion of biomass through the variations on pH, which may also prompt the decomposition of glucose derived from lignocellulosic biomass into FA, further experiments were performed using glucose as the model organic reductant and different bases: NaOH and phosphate buffer.

In a previous work,^4^ these experiments were performed using NaOH as a base, and the main findings obtained in that previous work are summarized here to provide context for the new results obtained with the phosphate buffer. Figure S2 presents the results obtained with different concentrations of NaOH, both in the presence and absence of NaHCO_3_. As presented in this figure, in experiments without NaHCO_3_, the addition of NaOH, and therefore the increase in pH to alkaline conditions, promotes the yield to FA, indicating that the conversion of glucose to FA is favored by alkaline conditions. On the other hand, the yields to FA in the presence of NaHCO_3_ are consistently higher than the yields without this compound as a consequence of NaHCO_3_ being an additional source of FA in these experiments. However, this increase in the total yield decreases as the concentration of NaOH is increased, and therefore pH is increased, and it is completely canceled in the experiment performed with a higher concentration of NaOH of 2 mol/L, in which the reaction with NaHCO_3_ produces the same yield as the reaction without it. This result is a consequence of the shift of the NaHCO_3_ acid/base equilibrium toward carbonate, which as observed in previous works is a non-reacting species.^6,30^

**Figure 4:**
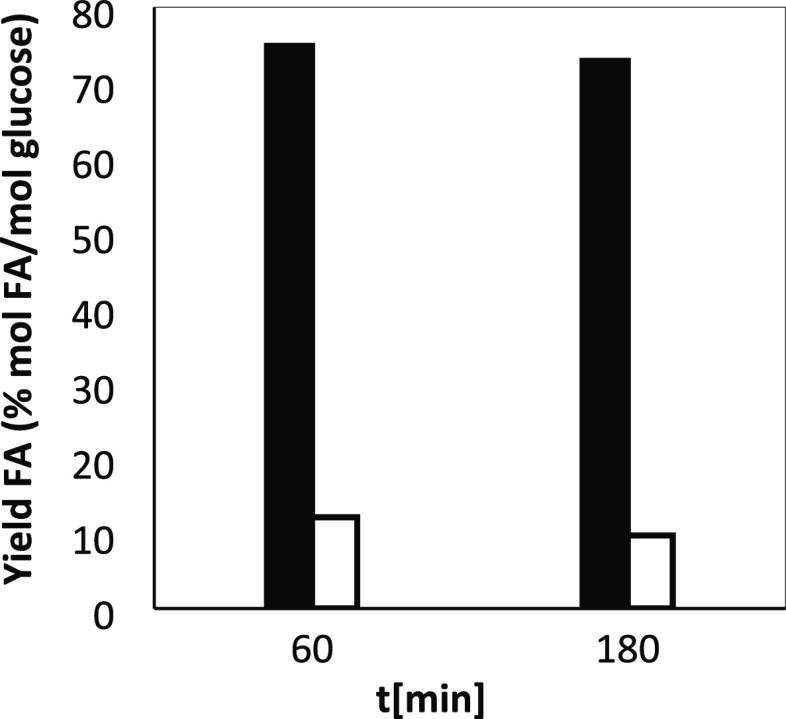
Effect on the yield to formic acid using 0.05 M of glucose as a reductant in a phosphate buffer solution at 300 °C during 60 and 180 min. (Black bars): reactions performed with 0.50 M NaHCO3 and (clear bars): reactions performed without NaHCO3.

It is thus observed that alkaline conditions are favorable for the oxidation of biomass toward acids but impair the conversion of CO_2_ dissolved in aqueous solutions as bicarbonate/carbonate. As in these experiments with NaOH, pH conditions are considerably different from experiments without this strong base, to further discriminate between the effect of NaHCO_3_ as a base that modifies the pH and as an oxidizing reagent, further experiments were performed in this work using a phosphate buffer (0.05 M NaH_2_PO_4_ and 0.50 M Na_2_HPO_4_) in a 0.05 M glucose solution that enables fixing a pH of 8, similar to the value provided by the buffer effect of bicarbonate. Experiments were performed with and without addition of 0.50 M NaHCO_3_ to isolate the influence of the buffer effect also produced by NaHCO_3_ on the conversion of glucose to FA from the reduction of NaHCO_3_. Results, presented in Figure 4, show that a low yield of formic acid was produced when no NaHCO_3_ was added, whereas the addition of NaHCO_3_ resulted in a greater production of formic acid, reaching a yield to formic acid of 75 and 73% after 60 and 180 min of reaction, respectively. This result demonstrates that the synergistic effect of NaHCO_3_ on the conversion of biomass is not related to its effect on pH but rather to its role as a second carbon source that is converted into FA and as an oxidant that promotes the conversion of biomass derivatives into organic acids.

### Characterization of Untreated Biomass and Solid Residue after Hydrothermal Treatment

The physical surface structure of untreated biomass and the solid residue recovered after the reaction were studied throughout SEM, FT-IR spectroscopy, and elemental analysis. At a macroscopic level, the differences of the hydrothermal treatment with and without NaHCO_3_ are evident, as shown in Figure S3. The solid residues remaining after hydrothermal treatment have a darker color and a more fragile structure than the starting material. These changes are more marked when adding NaHCO_3_. In the case of pine needles, they lose a large proportion of their mass.

**Figure 5:**
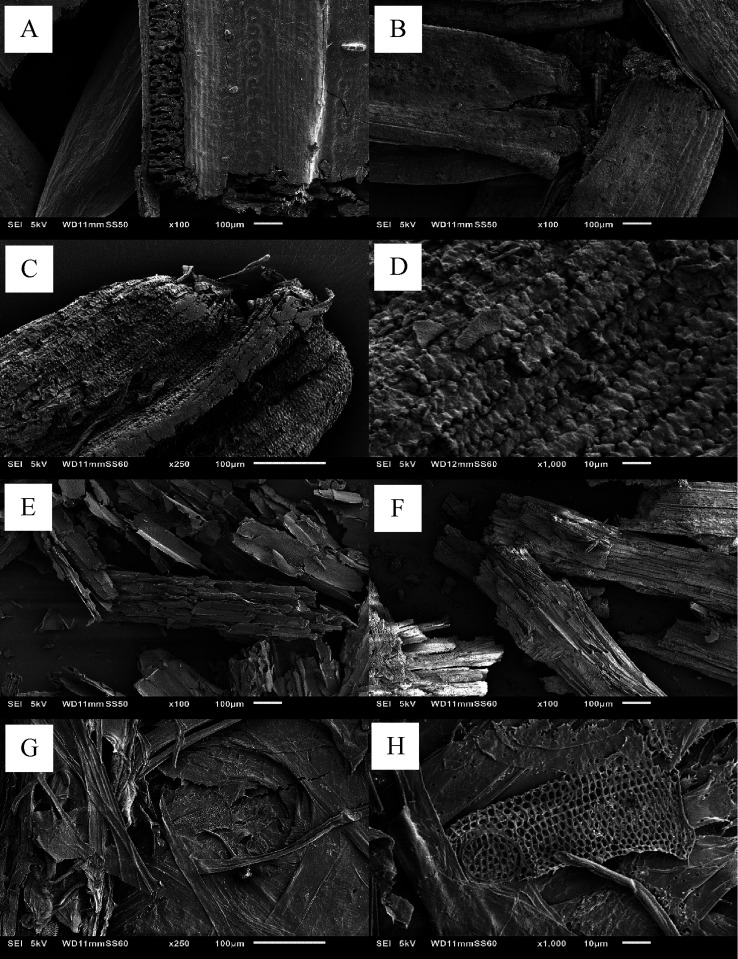
SEM images of (A–D) pine needles and (E–H) sugarcane bagasse solid samples. Three different conditions are displayed for both types of biomass: (A, E) untreated biomass, (B, F) hydrothermal treatment without NaHCO_3_ at 250 °C for 30 min, and (C, D, G, H) hydrothermal treatment with NaHCO_3_ at 250 °C for 30 min.

SEM images of PN and SB, both untreated and after the hydrothermal processing, are shown in Figure 5. In both cases, significant changes in the structure are observed. Hydrothermal treatment of pine needles without NaHCO_3_ (Figure 5B) resulted in a solid residue with a rougher surface in comparison to the untreated pine needles, which have a smoother surface (Figure 5A). The addition of NaHCO_3_ to the hydrothermal process resulted in a further roughening of the surface morphology (Figure 5C,D). These differences could be explained by the degradation of extracuticular wax present on the surface of pine needles; this degradation is incomplete when water alone is used but it is much more extensive when NaHCO_3_ is added,^31^ which is further evidence of the role of bicarbonate in the liquefaction of biomass indicated in the previous section. This is consistent with the limited extent of liquefaction achieved in the former scenario where these waxes may hinder the extraction of hydrocarbons. Closer inspection of the structures shown in Figure 5C reveals elongated independently arranged structures (Figure 5D), several tens of micrometers long. These may be the remains of cell walls, which have a comparable size.^32^ SEM images also show the superficial structural differences between pine needles and sugarcane bagasse (Figure 5E), the latter exhibiting a layered surface. After hydrothermal treatment, sugarcane bagasse residues have an irregular surface, either with or without the addition of NaHCO_3_. The solid residue from the hydrothermal treatment without NaHCO_3_ (Figure 5F) shows again a rougher surface, while the addition of bicarbonate appears to result in the collapse of the layered structure. Also, numerous net-type structures (Figure 5H,G), absent in the starting material, have been observed. These “nets” are hypothesized to be lignin domains in biomass, which remain unextracted upon hydrothermal treatment and are revealed due to the high level of degradation achieved. Similar resilient domains also appear to be present in the pine needles’ cell walls as their degradation is not uniform but is arranged in alternate sections.

**Figure 6:**
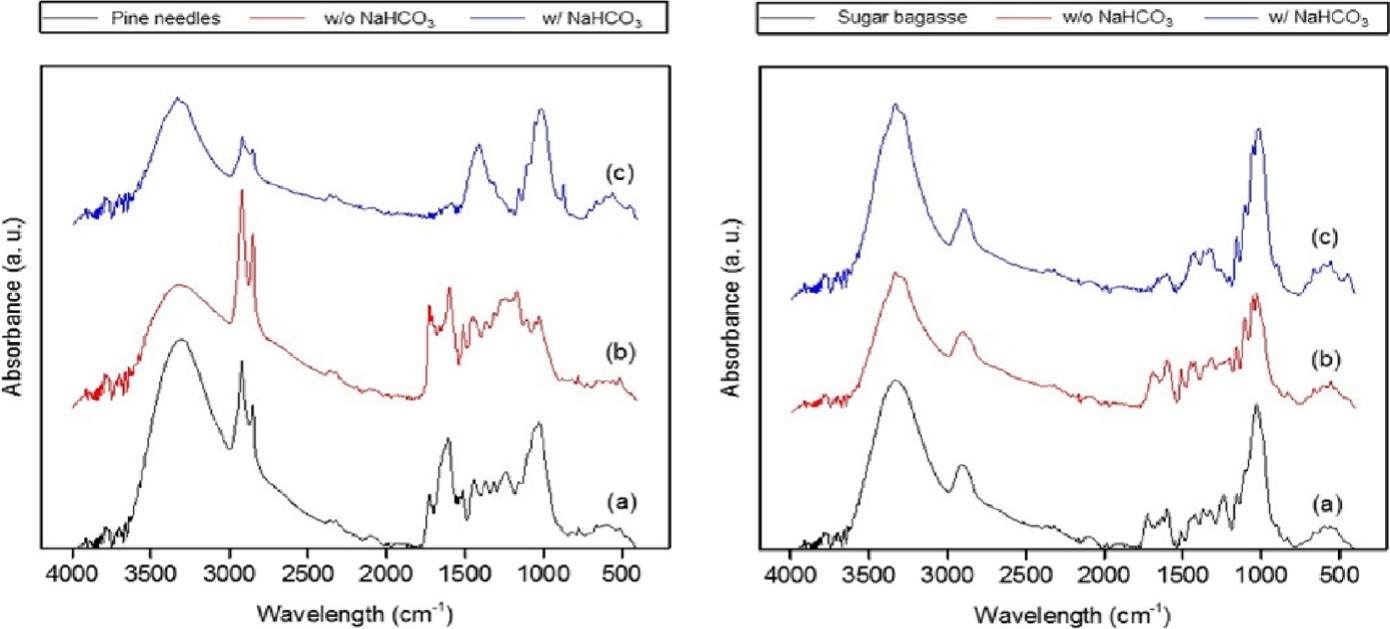
FT-IR spectra of untreated and hydrothermally treated PN (left) and SB (right). In both graphs: (a) untreated biomass, (b) hydrothermal treatment without NaHCO_3_ at 250 °C for 30 min, and (c) hydrothermal treatment with NaHCO_3_ at 250 °C for 30 min.

FT-IR spectra of untreated PN (Figure 6, left) and SB (Figure 6, right) show different bands related to lignocellulosic biomass. For example, bands at 3400 and 2920 cm^–1^ are assigned to −OH stretching and −CH_2_ stretching, respectively; these moieties are present in hemicellulose, cellulose, and lignin. Strong bands between 1000 and 1100 cm^–1^ are associated with C–O bonds in cellulose and hemicellulose, while bands between 1400 and 1800 cm^–1^ are characteristic of the aromatic C=C bending vibrations of lignin.^33,34^

Differences between untreated PN and SB are observed due to the differing composition of the two types of biomass.^25,26^ Notably, the spectral region assigned to vibrations characteristics of lignin (1400–1800 cm^–1^) is greater in intensity in the case of pine needles, relative to −OH (∼3400 cm^–1^) and C–O (∼1000 cm^–1^) regions, which can be related to hemicellulose and cellulose fractions. This observation supports the results described above: due to their greater lignin content, pine needles contain a lower proportion of readily available biomass hydrocarbons in the form of cellulose and hemicellulose, and hence a lower fraction of PN undergoes liquefaction when compared to SB.

Differences between untreated biomass and residues resulting from hydrothermal reactions can be observed in the obtained FT-IR spectra. However, their interpretation is hindered by the complexity of the samples. Spectra from the residue obtained from hydrothermal treatment of PN and SB without NaHCO_3_ show a relative decrease in the characteristic bands of carbohydrates with respect to those of lignin, which indicates the extraction of the former with lignin remaining preferentially in the solid. Using NaHCO_3_ in the hydrothermal treatment of pine needles resulted in a very different composition of the solid residue. It seems that, in addition to the extraction of carbohydrates, bicarbonate has affected further changes in the chemical structure that have even influenced the lignin component, evidenced by the significant reduction in intensity of the bands between 1400 and 1800 cm^–1^. It may be possible that these differences are due to hydrolysis reactions caused by an increase in pH.^35,36^

Table 1 shows the CHNS elemental analysis of the starting biomass and residues after hydrothermal treatment with and without bicarbonate. It should be noted that, whereas in all samples no S was detected, the content of the other elements in the samples differs significantly. The proportion H/C and O/C decreases upon hydrothermal treatment, which can be explained by the extraction of cellulose and hemicellulose, leaving mainly lignin in the residue. However, when bicarbonate is added, other reactions also occur, with the trends for H/C and O/C reversed, being higher than those of fresh biomass, typical of these transformations.^31^ It is proposed that the alkaline pH is promoting hydrolysis reactions within the solid matrix, which is consistent with the results obtained in FT-IR analysis. It can also be noted that the content of N also decreases upon hydrothermal treatment, and it is eliminated when adding bicarbonate, indicating that proteins are also being extracted from the solid matrix.

**Table 1:** Elemental Microanalysis for the Starting Biomass and the Residues after Treatment with and without NaHCO_3_^a^^,^^c^

	%N	%C	%H	H/C	%O^b^	O^b^/C
fresh PN	1.4	47.7	6.0	1.5	44.9	0.7
PN without NaHCO_3_	0.9	54.3	5.9	1.3	38.9	0.5
PN with NaHCO_3_	0.0	40.5	5.3	1.6	54.3	1.0
fresh SB	0.4	42.9	5.9	1.7	50.8	0.9
SB without NaHCO_3_	0.1	50.8	5.7	1.3	43.4	0.6
SB with NaHCO_3_	0.00	41.5	5.9	1.7	52.6	0.9

a PH = pine needles, SB = sugarcane bagasse.

b Oxygen content was estimated by difference.

c No S was detected in the samples.

## Conclusions

In this work, the hydrothermal biomass liquefaction and the hydrothermal NaHCO_3_ transformation into the sodium salt of formic acid (FA) have been combined in a one-pot reaction, without any previous purification of reductants. The addition of NaHCO_3_ resulted both in an increase in total dissolved organic carbon and in FA production, compared to results obtained without bicarbonate. Thus, the addition of NaHCO_3_ to the hydrothermal treatment of lignocellulosic residues prompts the formation of FA, contributing to the increase in the yield and selectivity toward important platform chemicals. The highest yield to FA (10% wt/wt) was achieved by using 5.0 g/L SB in 50 mL of an aqueous solution of 42.0 g/L NaHCO_3_ at 250 °C with a reaction time of 180 min. In agreement with previous works, the optimal yield to FA was obtained at long reaction times and at high temperatures using an excess of NaHCO_3_. The results suggest that the first reaction step is cellulose and hemicellulose extraction, subsequently followed by simultaneous reduction of NaHCO_3_ and biomass degradation, as formic acid, acetic acid (AA) and lactic acid (LA) are obtained. The complexity of the raw biomass and the complexity of the reaction mechanisms, not fully understood yet, are limitations for the selectivity of the reaction, which preferentially produce FA, AA, and LA but also smaller amounts of other organic compounds. Liquefaction of the structure of biomass is not homogeneous as different domains are degraded preferentially and other biological structures, such as extracuticular waxes that may play a role in the process.

With these results, this study sets a new strategy for CO_2_ and residual biomass valorization to produce value-added chemicals and renewable fuels, using only water as a solvent, in a simultaneous reaction that may simplify the process by eliminating or facilitating different steps (e.g., milling, drying, and liquefactions). The optimization of the selectivity of the reaction or the development of an efficient method for fractionating the main reaction products (formic, acetic, and lactic acid) is important challenges for the further development of this technology.

## Supporting Information Available

List of reagents used as HPLC standards, chromatogram of the reaction effluent, effect of the addition of NaOH on reaction yields, and photographs of solid samples before and after the hydrothermal treatment with and without NaHCO_3_ (PDF)
