# Supplementary material for: Synergistic Hydrothermal Conversion of Aqueous Solutions of CO2 and Biomass Waste Liquefaction into Formate
Source: ACS Sustain Chem Eng. 2022 Dec 2;10(50):16948–57. doi: 10.1021/acssuschemeng.2c06218 (PMC9769105; doi:10.1021/acssuschemeng.2c06218)
Supplement: Supplementary file 1 — sc2c06218_si_001.pdf [file sc2c06218_si_001.pdf]

Supporting Information

Synergistic hydrothermal conversion of aqueous solutions of CO_2 and biomass waste liquefaction into formate

María Andérez-Fernández[a], Eduardo Pérez[b], Ángel Martín[a], James McGregor[c], María Dolores Bermejo[a]*

[a]Grupo de Tecnologías a Presión (PressTech), Instituto de Bioeconomía de la Universidad de Valladolid (BioEcoUVa), Departamento de Ingeniería Química y Tecnologías del Medio Ambiente, Escuela de Ingenierías Industriales, Universidad de Valladolid, 47011, Valladolid, Spain

[b]Departamento de Química Física. Facultad de Químicas. Universidad Complutense de Madrid. Avda Complutense s/n 28040 Madrid, Spain

[c]University of Sheffield, Department of Chemical and Biological Engineering, Sheffield S1 3JD, UK

Tel: +34 983423167, e-mail: mamaan@iq.uva.es (Á. Martín)

Number of pages: 5

Number of figures: 3

Number of tables: 0

Number of schemes: 0

EXPERIMENTAL SECTION

Materials

For standards for HPLC analysis, the following chemicals where used: glycerol (99.5%), n-propanol (> 99.7%), glyceraldehyde (90%), glycolaldehyde dimer (99%), pyruvaldehyde (40%), 5-HMF (99%), furfural (99%), fructose (99%), rahmnose (99%), resorcinol (99%), glucuronic acid (> 98%), oxalic acid (≥ 99%), acrylic acid (99%), d-glyceric acid calcium salt (99%), cathecol (99%), D-(+)-glucose (99%) and guaiacol (> 99%) were bought to Sigma Aldrich. Ethanol (EtOH, 99.5%), acetone (99.5%), isopropanol (iPrOH, 99.9%), formaldehyde stabilized in MeOH (37-38% wt/wt), $Na_2HPO_4·7H_2O$ (99%) and propanoic acid (99%) were acquired from Panreac. Formic acid (98%) and galacturonic acid (≥ 97%) were purchased from Fluka. Methanol (MeOH, 99.99%) was obtained from Fisher Scientific. Ethylenglycol (≥ 99.5%) was obtained from Merck. All the chemicals were used without further treatment.

RESULTS AND DICUSSION

Characterization of reaction effluent

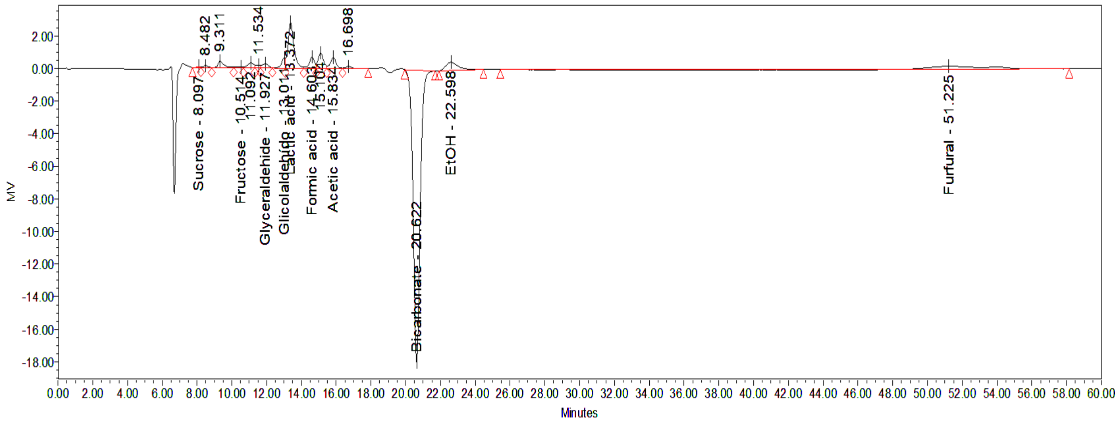

Figure S1. Chromatographic analysis of the reaction effluent, experiment performed with 0.1 g of sugar bagasse as reductant at 250^{0}C and 30 min.

Influence of the alkaline medium in the reaction

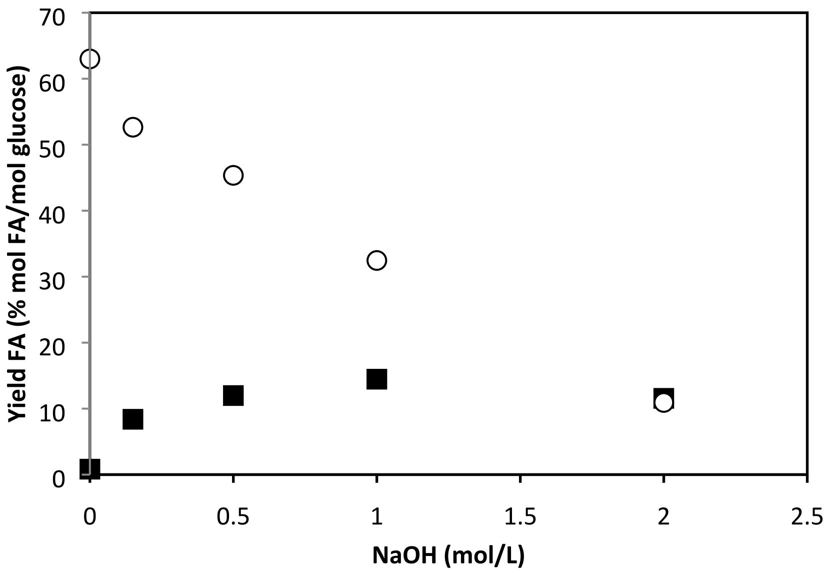

Figure S2. Effect of NaOH concentration on the yield to formic acid using glucose as reductant at 300ºC for 180 min. (■): in absence of $NaHCO_3$; (○): in presence of 0.50M $NaHCO_3$. Reprinted (Adapted or Reprinted in part) with permission from Andérez-Fernández, M.; Pérez, E.; Martín, A.; Bermejo, M. D. Hydrothermal CO_2 Reduction Using Biomass Derivatives as Reductants. The Journal of Supercritical Fluids 2018, 133, 658–664. Copyright 2018 Elsevier.

Effect of hydrothermal treatment with and without $NaHCO_3$

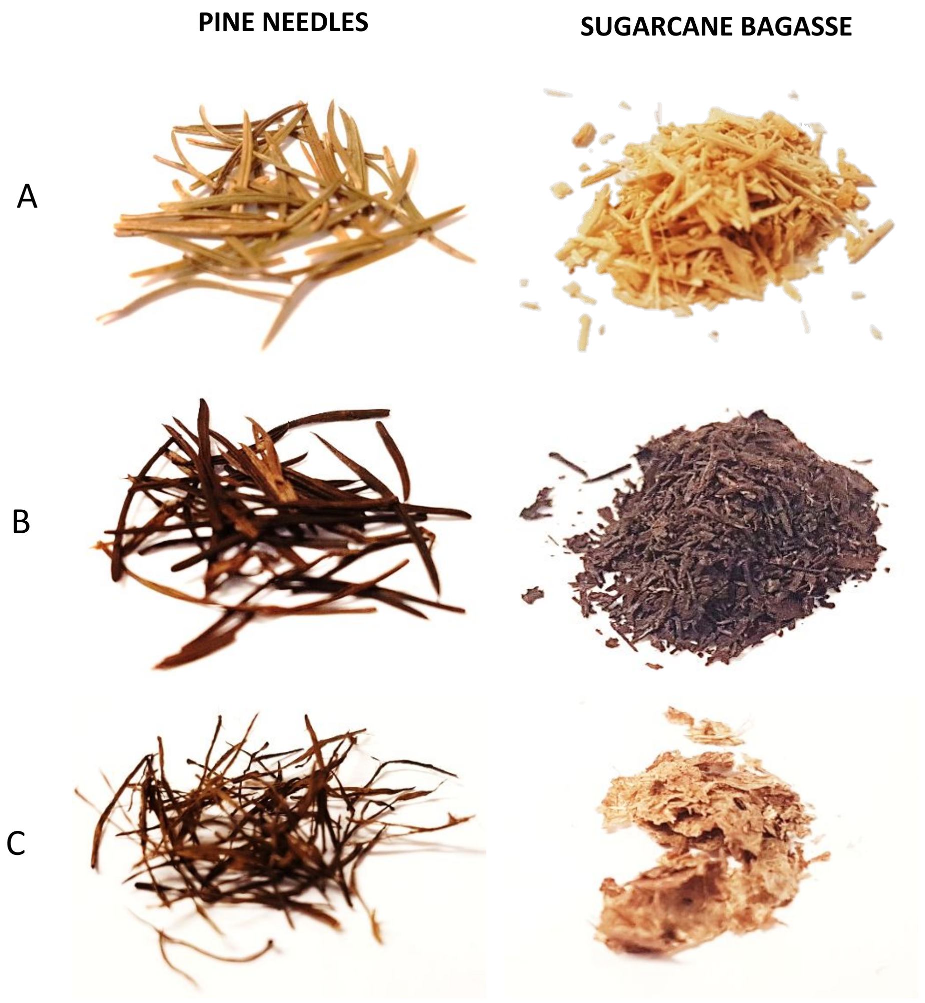

Figure S3. Solid samples of pine needles and sugarcane bagasse. a) Untreated biomass; b) hydrothermal treatment without NaHCO3 at 250 ˚C for 30 min; and c) hydrothermal treatment with NaHCO3 at 250 ˚C for 30 min.
